# New Mutations in *cls* Lead to Daptomycin Resistance in a Clinical Vancomycin- and Daptomycin-Resistant *Enterococcus faecium* Strain

**DOI:** 10.3389/fmicb.2022.896916

**Published:** 2022-06-21

**Authors:** Weiwei Li, Jiamin Hu, Ling Li, Mengge Zhang, Qingyu Cui, Yanan Ma, Hainan Su, Xuhua Zhang, Hai Xu, Mingyu Wang

**Affiliations:** ^1^State Key Laboratory of Microbial Technology, Microbial Technology Institute, Shandong University, Qingdao, China; ^2^Division of Science and Technology, Ludong University, Yantai, China; ^3^Laboratory Medicine Center, The Second Hospital of Shandong University, Jinan, China

**Keywords:** vancomycin-resistant enterococci, *Enterococcus faecium*, daptomycin resistance, vancomycin resistance, membrane surface charge, *gdpD*, *cls*

## Abstract

Daptomycin (DAP), a last-resort antibiotic for treating Gram-positive bacterial infection, has been widely used in the treatment of vancomycin-resistant enterococci (VRE). Resistance to both daptomycin and vancomycin leads to difficulties in controlling infections of enterococci. A clinical multidrug-resistant *Enterococcus faecium* EF332 strain that shows resistance to both daptomycin and vancomycin was identified, for which resistance mechanisms were investigated in this work. Whole-genome sequencing and comparative genomic analysis were performed by third-generation PacBio sequencing, showing that *E. faecium* EF332 contains four plasmids, including a new multidrug-resistant pEF332-2 plasmid. Two vancomycin resistance-conferring gene clusters *vanA* and *vanM* were found on this plasmid, making it the second reported vancomycin-resistant plasmid containing both clusters. New mutations in chromosomal genes *cls* and *gdpD* that, respectively, encode cardiolipin synthase and glycerophosphoryl diester phosphodiesterase were identified. Their potential roles in leading to daptomycin resistance were further investigated. Through molecular cloning and phenotypic screening, two-dimensional thin-layer chromatography, fluorescence surface charge test, and analysis of cardiolipin distribution patterns, we found that mutations in *cls* decrease surface negative charges of the cell membrane (CM) and led to redistribution of lipids of CM. Both events contribute to the DAP resistance of *E. faecium* EF332. Mutation in *gdpD* leads to changes in CM phospholipid compositions, but cannot confer DAP resistance. Neither mutation could result in changes in cellular septa. Therefore, we conclude that the daptomycin resistance of *E. faecium* EF332 is conferred by new *cls* mutations. This work reports the genetic basis for vancomycin and daptomycin resistance of a multidrug-resistant *E. faecium* strain, with the finding of new mutations of *cls* that leads to daptomycin resistance.

## Introduction

*Enterococcus faecium*, a Gram-positive coccus, is an important nosocomial pathogen that can cause fatal bacteremia and myocarditis (Arias and Murray, 2012). The emergence of multidrug-resistant (MDR) *E. faecium*, particularly vancomycin-resistant enterococci (VRE), led to high morbidity and mortality in hospitalized patients (Tran et al., 2013a, 2015; Herc et al., 2017; Lebreton et al., 2018). To make matters worse, the horizontal transfer of antibiotic resistance genes (ARGs) directly led to the rapid increase in VRE. In recent decades, VRE was gradually recognized as a major cause of MDR hospital infection in many countries (Uttley et al., 1988; Cattoir and Giard, 2014). A rapid increase of VRE undoubtedly brings more difficulties for the treatment of *E. faecium* infections (Depardieu et al., 2007; Lebreton et al., 2018). Initially, linezolid and quinupristin–dalfopristin were used to treat VRE infections, but their use was hampered by their strong adverse drug effects (Carver et al., 2003; Hogan et al., 2010; Matsumoto et al., 2010). By contrast, daptomycin (DAP) was generally considered to be a safer antimicrobial agent and gradually became the front-line drug for the patients who have VRE infections (Chow et al., 2016; Gonzalez-Ruiz et al., 2016; Herc et al., 2017; Suleyman et al., 2017).

Daptomycin is a cyclic lipopeptide antibiotic produced by *Streptomyces roseosporus* (Heidary et al., 2018), which has a special mechanism that binds to Ca^2+^ and inserts into the cell membrane (CM) causing leakage of intracellular ions and ATP, thus killing bacteria (LaPlante and Rybak, 2004; Heidary et al., 2018). Unfortunately, the widespread use of daptomycin resulted in the emergence of daptomycin non-susceptible VRE (DNVRE), which undoubtedly further reduced the option of treatment for VRE infections (Munoz-Price et al., 2005; Lellek et al., 2015; Chow et al., 2016; Hussain et al., 2016; Herc et al., 2017). Multiple studies suggested that the mechanism of daptomycin resistance is mainly related to mutations in chromosomal genes, including *mprF*, *gdpD*, *yycG*, *rpoB*, *rpoC*, *pgsA*, *cls*, *liaFSR*, etc. (Fischer et al., 2011; Tran et al., 2013a,b; Heidary et al., 2018). With the increase of reports on DNVRE, a series of investigations were carried out to try to find the resistance mechanisms of daptomycin and effective applications of DAP (Munoz-Price et al., 2005; Lellek et al., 2015; Chow et al., 2016; Hussain et al., 2016; Suleyman et al., 2017). However, the mechanism of DAP resistance has not been fully elucidated: One view is that DAP is diverted from effective targets at the septum by redistribution of anionic phospholipids; another view is that resistant bacteria achieve electrostatic repulsion to DAP by reducing the negative surface charge of CM (Heidary et al., 2018). In summary, the reports on DAP resistance mainly focus on the interaction between CM and DAP–Ca^2+^ complex.

In view of this, in the present study, a clinical multidrug-resistant *E. faecium* from a hospital showing daptomycin resistance and high-level vancomycin resistance was identified. Whole-genome sequencing and subsequent mechanistic investigations were performed, hoping to find the answers to the following questions: (1) the genetic basis of the multidrug resistance; (2) the risk of multiple resistance transmission; and (3) the mechanism of daptomycin resistance.

## Materials and Methods

### Strains

*Enterococcus faecium* EF332 strain used in this study was isolated in the Laboratory Medicine Center of the Second Hospital of Shandong University. The laboratory maintains an opportunistic pathogen library isolated from patient samples and routinely screens them for possible new findings. The strain was identified by the analysis of 16S rDNA sequence.

### Antibiotic Susceptibility Tests

Antibiotic susceptibility tests were conducted according to CLSI/EUCAST guidelines (EUCAST, 2017; CLSI, 2018). The tested antibiotics include: penicillin (PEN), ampicillin (AMP), ciprofloxacin (CIP), gatifloxacin (GAT), chloramphenicol (CHL), tetracycline (TET), tigecycline (TGC), fosfomycin (FOF), erythromycin (ERY), linezolid (LZD), rifampicin (RIF), vancomycin (VAN), and daptomycin (DAP). K-B Disk diffusion assays were performed as previously documented (Vading et al., 2011). Agar dilution method (for FOF) and broth microdilution method (for the rest antibiotics) were adopted to determine MICs of antibiotics. *Staphylococcus aureus* ATCC 25923 and *Enterococcus faecalis* ATCC 29212 were used as control strains for disk diffusion and dilution method, respectively, according to CLSI and EUCAST standards (EUCAST, 2017; CLSI, 2018).

### Extraction and Sequencing of Genomic DNA

The genomic DNA of *E. faecium* EF332 and constructed strains (29212-pDL278, EFDO-*cls*, EF332-*cls*) was extracted following previous reported protocol (Qian and Zhao, 2014), and the purity and integrity of the DNA were confirmed by agarose gel electrophoresis. DNA samples of *E. faecium* EF332 were constructed into a 10-kb SMRTbell DNA library, and PacBio single-molecule sequencing was used to obtain at least 50× of sequencing data. In addition, a 350-bp small fragment library was constructed, and Illumina NovaSeq PE150 platform was used for paired-end sequencing to obtain at least 100× clean data for auxiliary assembly. DNA samples of constructed strains were used as templates for quantitative polymerase chain reaction (qPCR) to detect the relative copy number of plasmids.

### Conjugation and Transformation of Plasmid

To verify the transferability of plasmid pEF332-2, we conducted conjugation and transformation experiments. A chloramphenicol-resistant and vancomycin-sensitive *E. faecalis* 3–147 strain was used as the recipient for conjugations, and vancomycin-sensitive *E. faecalis* ATCC 29212 was used as recipient for transformation. Transconjugants were identified on the plates containing 32 μg/ml of vancomycin and 50 μg/ml of chloramphenicol, and transformants were identified on the plates containing 32 μg/ml of vancomycin.

### Acquisition, Cloning, and Transformation of Presumed DAP Resistance Genes

Genomic DNA of *E. faecium* EF332 strain was used as template to amplify the presumed DAP resistance genes *cls* and *gdpD* by PCR using Phanta super-fidelity DNA polymerase (Vazyme Biotech Co. Ltd. Nanjing, China). All the PCR products were analyzed by 1% agarose gel electrophoresis and purified by DNA clean-up kit (TIANGEN, Beijing, China).

All primer sequences are shown in Supplementary Table 1, in which the cleavage sites of BamHI and SalI were added to the 5′ ends for the next step of cloning. The shuttle plasmid pDL278 with spectinomycin resistance was used for molecular cloning (Genbank accession number AF216802.1). The *cls*, *gdpD* fragments and pDL278 vector, were digested by BamHI and SalI endonuclease, respectively, and then, the gene fragments were ligated to pDL278 vector by T4 DNA ligase to generate recombinant shuttle plasmids (pDL278-*cls*, pDL278-*gdpD*). These recombinant plasmids were transferred into *Escherichia coli* DH5α chemically competent cells, respectively, according to the manufacturer’s protocols, and Luria-Bertani (LB) agar plates with 500 mg/L spectinomycin were used to select for positive transformants. The plasmids of the positive clones were extracted and transformed into the electroporation competent cells of *E. faecalis* ATCC 29212, and tryptic soy broth (TSB) agar plates with 1,000 mg/L spectinomycin were used to select for positive transformants. The successfully transferred gene fragments were validated by Sanger sequencing using primer M13 (as shown in Supplementary Table 1). Finally, we obtained the EF332-*cls* and EF332-*gdpD* strains for downstream analysis. A summary of constructed strains is shown in Table 1 for easier comparison.

**Table 1:** Constructed strains in this study.

Strain	Description
EF332-*cls*	*Enterococcus faecalis* ATCC 29212 harboring plasmid pDL278 that carries *cls* gene originated from *E. faecium* EF332
EFDO-*cls*	*Enterococcus faecalis* ATCC 29212 harboring plasmid pDL278 that carries site-directed mutated *cls* gene originated from *E. faecium* DO
EF332-*gdpD*	*Enterococcus faecalis* ATCC 29212 harboring plasmid pDL278 that carries *gdpD* gene originated from *E. faecium* EF332
EFDO-*gdpD*	*Enterococcus faecalis* ATCC 29212 harboring plasmid pDL278 that carries site-directed mutated *gdpD* gene originated from *E. faecium* DO
29,212-pDL278	*Enterococcus faecalis* ATCC 29212 harboring shuttle plasmid pDL278

### Site-Directed Mutagenesis

In order to further confirm the role of mutations in *cls* and *gdpD* genes, site-directed mutagenesis was performed using overlap extension PCR (SOE-PCR; Ho et al., 1989). The *cls* gene was divided into four fragments at three mutated sites, and *gdpD* gene was divided into two fragments at one mutated site. The primers used are shown in Supplementary Table 1.

The SOE-PCR process consisted of two consecutive reactions. First-round PCR: Genomic DNA of *E. faecium* EF332 was used as a template to obtain the corresponding gene fragments. PCR products obtained were purified using the DNA purification kit (TIANGEN, Beijing, China). Second-round PCR: The purified PCR products obtained from the previous step were used as templates, and corresponding primers were added to the reaction system for amplification.

These point mutations led to the amino acid substitutions T269I, V203I, T298S in Cls, and S201P in GdpD. Plasmids carrying these mutated gene fragments were transformed to obtain EFDO-*cls* and EFDO-*gdpD* strains by the method described above.

### Analysis of Membrane Surface Charge

Poly-_L_-lysine conjugated to fluorescein isothiocyanate (PLL:FITC) was used to assay cell surface charges according to previous studies (Prater et al., 2021). In brief, the strains grew until OD_600_ reached 0.5 in TSB. Cells were then washed three times with sterilized HEPES buffer (20 mM, pH 7.0) and diluted to OD_600_ of 0.1. Cells were incubated with 50 μg/ml PLL:FITC (final concentration) at room temperature with shaking for 10 min and were then washed once using HEPES buffer to remove unbound PLL:FITC. Treated cells were spread on poly-_L_-lysine-treated glass slides, followed by the addition of 20 μl fluorescent anti-quenching agent. Fluorescence images were observed under NIKON Ti-E inverted fluorescence microscope (Nikon Instruments Co., LTD, Japan) and quantified by ImageJ, with duplicates per strain.

### Observation of Cardiolipin Distribution on CM

The fluorescent probe 10-*N*-nonyl acridine orange (NAO) specifically binds to cardiolipin, and the change of cardiolipin distribution caused by *cls* mutation can be determined by observing the NAO fluorescence distribution on the membrane (Mileykovskaya et al., 2001). NAO fluorescence assay was performed according to previous studies (Tran et al., 2013b). In brief, the bacteria were activated in TSB overnight and transferred to new TSB to grow to exponential phase (OD_600_ of ~0.5), after which NAO was added to the medium at a final concentration of 2.5 μM. The sample was oscillated at 100 rpm and 37°C for 4 h under dark conditions. The cells were washed three times (5,000 rpm, 3 min) with sterilized 0.9% saline and immediately mixed with 2× volume of fluorescent anti-quenching agent and fixed on the poly-_L_-lysine-treated glass slides. Fluorescence observation was performed using a laser scanning confocal microscope with 100× objective and airyscan detector (LSM880, ZEISIS, Germany).

### CM Lipid Analysis

Membrane lipids were extracted using previous methods (Komagata and Suzuki, 1988). The lipid components of tested strains were separated by two-dimensional thin-layer chromatography (2D-TLC, Silica 60 F254 TLC plates; Merck) and stained with phosphomolybdic acid (blue for all lipids) and ninhydrin (red for lipids with amino groups), respectively, with three replicates for each strain. The first dimension was developed with chloroform/methanol/water (65:25:4, by volume), and the second dimension was developed with chloroform/acetic acid/methanol/water (80:15:12:4, by volume). The dried TLC plates were heated for 15 min in a 110°C oven to find phospholipid composition.

### Transmission Electron Microscopy

Bacteria were inoculated in 30-ml TSB and grew to exponential phase by shaking at 160 rpm and 37°C. The cultures were centrifuged at 5,000 rpm for 3 min to pellet cells. Cells were fixed at 4°C for 1 h with 500 μl 2.5% glutaraldehyde, washed with PBS (pH 7.2, 0.01 M) for five times (20 min each), fixed with 1% osmium acid at 4°C for 1 h, and washed with PBS (pH 7.2, 0.01 M) for three times, 15 min each. The samples were sequentially dehydrated with 30, 50, 70, 90, and 100% ethanol (twice) and replaced twice by acetone, 15 min each time. Different proportions of embedding agent (EPON812: acetone =1:3,1:1,3:1) were used to penetrate for 1 h successively, and then, pure embedding agent (100% EPON812) was used for penetration overnight. The samples were embedded in EPON812 and then subjected to temperature-programmed curing for 48 h before ultra-thin section. The sections were stained with 2% uranium acetate solution for 20 min (in dark), washed with ddH_2_O three times for 5 min each, stained with 1% lead citrate solution for 10 min, and then washed with ddH_2_O three times for 5 min each. The prepared samples were observed by Focused Ion Beam-Scanning Transmission Electron Microscope (Crossbeam 550, ZEISIS, Germany).

### Quantitative Polymerase Chain Reactions

qPCRs were used to assay the copy numbers of pDL278 vector and variants in *E. faecium* strains (29212-pDL278, EFDO-*cls*, EF332-*cls*) to verify the stability of this vector. Plasmid levels were calculated using the 2^−ΔΔ*C*t^ method. 16S rDNA was used as the housekeeping gene. The primers used are listed in Supplementary Table 1. The qPCR system (20 μl) contains the following: 10 μl of 2× SYBR green premix pro taq HS premix (Accurate Biotechnology Co., Ltd., China), 7.8 μl dd H_2_O, 0.4 μl of forward and reverse primers each (10 μM), 0.4 μl of ROX reference dye (20 μM, Accurate Biotechnology Co., Ltd., China), and 1 μl template DNA (100 ng/μl). The qPCRs were performed with the following programs: denaturation at 95°C for 30 s, followed by 40 cycles of denaturation at 95°C for 5 s, annealing at 60°C for 30 s. Three biological replicates were performed for each sample, and each qPCR was conducted in triplicate using the ABI StepOnePlus system (Applied Biosystems Inc., Waltham, MA, United States).

### Survival Assay

Survival analysis was performed according to the previously published method to compare the survival of different strains under daptomycin stress (Grein et al., 2020). After the bacteria grew to exponential phase (OD_600_ = 0.5) in 5 ml Müller Hinton broth containing 0.05 g/L Ca^2+^ (CAMHB), 100 μl of the cultures were diluted 10^5^-fold. One hundred microliters of the diluted bacterial solution was inoculated on TSB agar plates (three replications) for CFU calculation. The remaining cultures were incubated with 32 μg/ml (4 × MIC) daptomycin (final concentration) at 37°C with shaking at 180 rpm for 1 h. During incubation, 100 μl cultures were taken every 15 min, diluted, and counted as described above. In order to avoid the influence of the change of bacterial solution volume during incubation, an equal volume of CAMHB was added after 100 μl bacterial solution was taken each time. Colony counts at different time points were recorded after overnight incubation at 37°C, and survival rate was calculated as the percentage of colony counts to those without treatment of daptomycin.

### Bioinformatics

Whole-genome sequencing reads were assembled into a preliminary genome using SMRT Link v5.1.0 software (Ardui et al., 2018), and arrow software was used to optimize the assembly results. The optimized assembly results were analyzed and compared with original data to discriminate chromosomes and plasmid sequences. Final circular genomes were obtained, followed by gene prediction using GeneMarkS v4.17 software (Besemer et al., 2001). Genomic Islands (GIs) were predicted using IslandPath-DIOMB v0.12 software (Hsiao et al., 2003). Prophage prediction was completed by online prediction server PHASTER (Zhou et al., 2011; Arndt et al., 2016). Functional annotation of coding genes was performed using GO (Ashburner et al., 2000), COG (Galperin et al., 2014), NR (Li et al., 2002), Pfam (Punta et al., 2011), TCDB (Saier et al., 2013), and SWISS-PROT (Bairoch and Apweiler, 2000) databases. ARG annotation used Resistance Gene Identifier (RGI) v5.1.0 software provided by CARD database (Jia et al., 2016). Multilocus sequence typing (MLST) and plasmid classification of *E. faecium* EF332 were performed using MLST^1^ and PlasmidFinder databases,^2^ respectively, provided by Center for Genomic Epidemiology. AlphaFold 2 software was used to construct Cls structure model (Jumper et al., 2021). Pfam database was used to predict Cls protein domain structure (Mistry et al., 2021), and DOG 2.0 software was used to draw the protein domain structure map (Ren et al., 2009). The relative intensity of fluorescence was quantified by ImageJ 1.52a, resulting in the mean of two biological duplicates, and the significance level was analyzed by Student’s *t*-test. The ggplot2 package in R Studio 1.3.929 software (based on R 3.6.3) was used to draw the box charts of fluorescence intensity, and the gggenes and ggplot2 packages were used to generate the gene structure diagrams.

## Results

### Isolation and Identification of a Vancomycin- and Daptomycin-Resistant *Enterococcus faecium* EF332 Strain

*Enterococcus faecium* EF332 strain used in this study was isolated in the Laboratory Medicine Center of the Second Hospital of Shandong University during routine screening of pathogens. Antimicrobial susceptibility tests were performed for this strain using 13 antibiotics in 10 classes by both Kirby–Bauer disk diffusion method and determination of minimum inhibitory concentration (MIC) levels. It was shown that *E. faecium* EF332 was resistant to most antibiotics (Table 2), suggesting that it is multidrug-resistant. A striking observation is that *E. faecium* EF332 is resistant to both last-line antibiotics against enterococcal infections: vancomycin and daptomycin (Bender et al., 2018). This unusual phenomenon drove us to further investigate the mechanisms underlying multidrug resistance of this strain, particularly for the two last-resort antibiotics vancomycin and daptomycin.

**Table 2:** Antibiotic susceptibility of *Enterococcus faecium* EF332.

Antibiotics class	Antibiotic	Inhibition zone (mm)	MIC (μg/ml)
Ansamycins	RIF	11 (R)	4 (R)
Macrolides	ERY	0 (R)	>256 (R)
Fluoroquinolones	CIP	0 (R)	32 (R)
	GAT	0 (R)	32 (R)
Phenicols	CHL	21 (S)	4 (S)
Tetracyclines	TET	10 (R)	32 (R)
	TGC	22 (S)	0.0375 (S)
Glycopeptides	VAN	0 (R)	128 (R)
β-lactams	PEN	0 (R)	>256 (R)
	AMP	0 (R)	>256 (R)
Lipopeptides	DAP	n.a.	8 (R)
Fosfomycins	FOF	34 (S)	64 (S)
Oxazolidinones	LZD	23 (S)	1 (S)

n.a.: K-B disk diffusion method is unavailable in both CLSI and EUCAST standards. RIF, rifampicin; ERY, erythromycin; CIP, ciprofloxacin; GAT, gatifloxacin; CHL, chloramphenicol; TET, tetracycline; TGC, tigecycline; VAN, vancomycin; PEN, penicillin; AMP, ampicillin; DAP, daptomycin; FOF, fosfomycin; LZD, linezolid; R, resistant; S, sensitive.

### Multidrug-Resistant Determinants of *Enterococcus faecium* EF332 Revealed by Whole-Genome Sequencing

To fully understand the genetic basis of multidrug resistance in *E. faecium* EF332, whole-genome sequences were obtained with third-generation PacBio sequencing accompanied by second-generation Illumina sequencing (The sequencing data were deposited in GenBank with accession numbers CP058891-CP058895). Full sequences for a circular chromosome and four plasmids were obtained. The size of the chromosome is 2,755,510 bp, similarly to previously sequenced *E. faecium* strains. The four plasmids, pEF332-1, pEF332-2, pEF332-3, and pEF332-4, are, respectively, 12,341, 87,675p, 28,308, and 203,652 bp long (Supplementary Figure 1; Supplementary Table 2). Multilocus sequence typing (MLST) result showed that *E. faecium* EF332 belongs to ST192, one type of the CC17 clone complex (clade A1), which is believed to have evolved in infections of hospital environment (Freitas et al., 2010).

**Figure 1:**
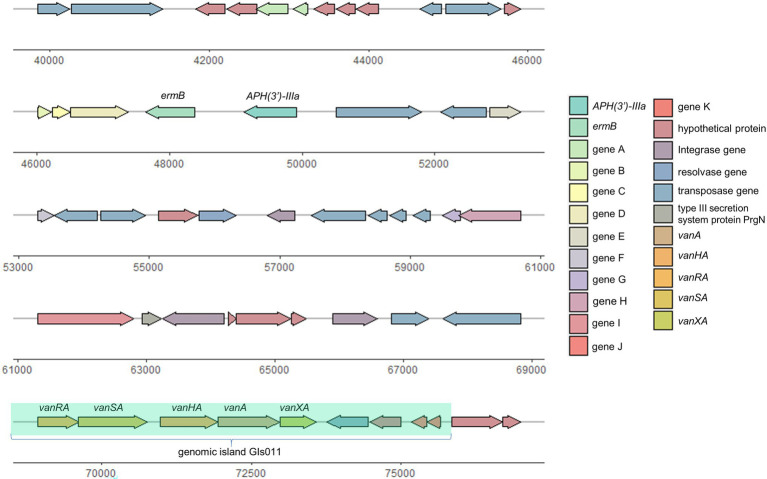
Genetic organization of prophage in pEF332-2.

**Figure 2:**
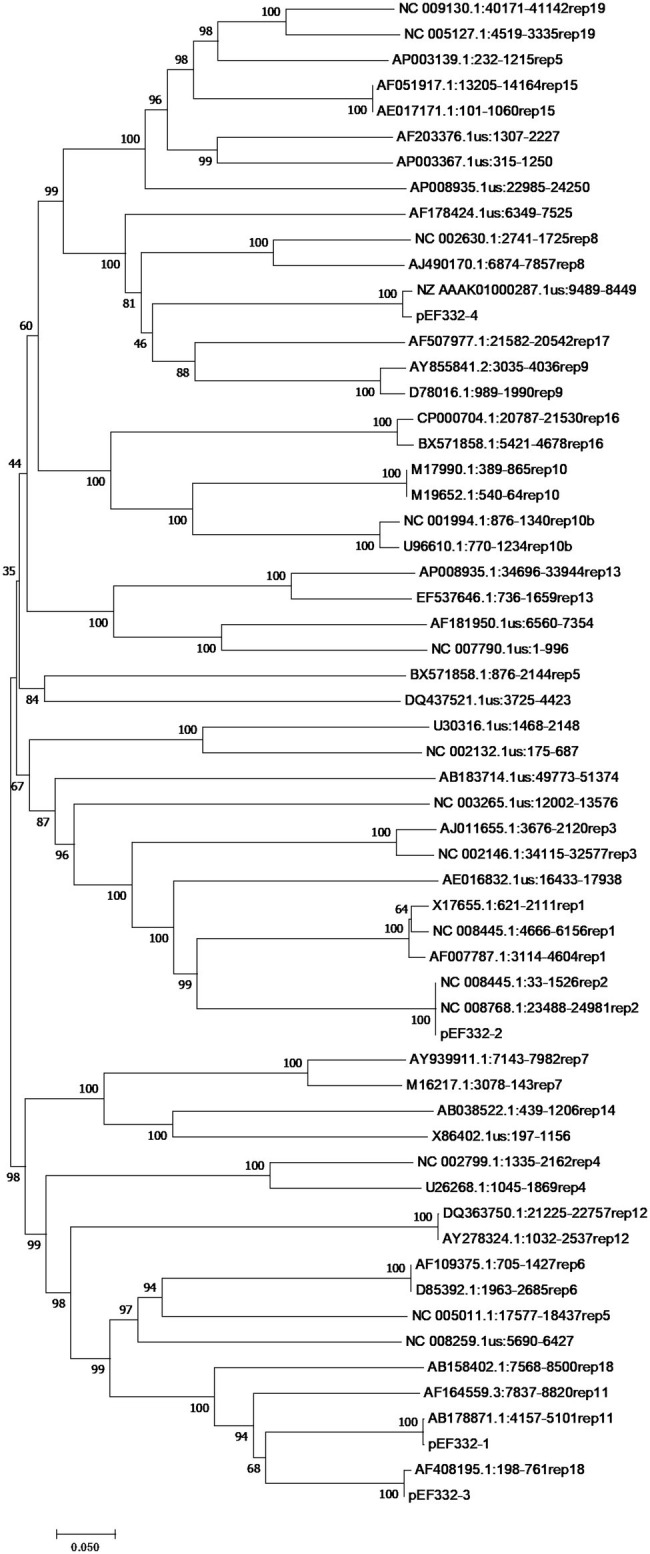
Phylogenetic analysis of *rep* genes of *Enterococcus faecium* EF332 plasmids. The bootstrap values are displayed on the branches with 1,000 times of replications. Bar, evolutionary distance.

With accurate gene prediction algorithms, a total of 3,121 genes were predicted to be encoded by *E. faecium* EF332, of which 2,735 are chromosome-borne and 386 are plasmid-borne (Supplementary Table 2). Annotation with Comprehensive Antibiotic Resistance Database (CARD) showed that 9, 0, 12, 0, and 2 ARGs were encoded on the chromosome, pEF332-1, pEF332-2, pEF332-3, and pEF332-4, respectively (Table 3; Supplementary Table 2). Determinants for all antibiotics were found, most of which are chromosome-borne. Only pEF332-2 and pEF332-4 are antibiotic-resistant plasmids. While pEF332-4 only carries ARGs for aminoglycosides and was previously found in *E. faecium* VRE1 (Sun et al., 2020), pEF332-2 is a new plasmid that carries both *vanA* and *vanM* gene clusters. To the best of our knowledge, plasmids carrying both *vanA* and *vanM* genes are very rare, and only one such plasmid (pELF1) has been found so far in Japan, which is associated with high vancomycin resistance and high risk of transmission (Hashimoto et al., 2019). pEF332-2 is therefore the second plasmid that bears both *vanA* and *vanM* clusters identified so far. This is also in consistency with the high level of vancomycin resistance (128 μg/ml) observed with *E. faecium* EF332. In addition, *vanA* gene clusters are encoded on a genomic island GIs011 that is also part of a multidrug-resistant prophage (Figure 1; Supplementary Table 3). This is a strong implication that these ARGs are acquired by horizontal gene transfer. Therefore, this new pEF332-2 plasmid is a strong vancomycin-resistant plasmid that also has a strong implication for ARG mobility. Further phylogenetic analysis of *E. faecium* EF332 plasmids and 55 other plasmids, along with subtyping of pEF332-2 with PlasmidFinder, suggests pEF332-2 belongs to type rep2 (Figure 2). Previous report suggested type rep2 plasmids originate from a variety of sources, including hospitals, poultry, and foods (Schwarz et al., 2001; Sletvold et al., 2007; Sun et al., 2020), suggesting that these plasmids may spread between multiple environments. Nevertheless, attempts to transfer pEF332-2 from the host *E. faecium* EF332 strain to another bacterium *via* conjugation or transformation were unsuccessful, showing the limited efficiency of horizontal gene transfer.

**Table 3:** Predicted antimicrobial resistance determinants in *Enterococcus faecium* EF332 with CARD.

Antibiotics class	Chromosome	pEF332-2	pEF332-4
Fluoroquinolones	*gyrA* mutation		
	*gyrB* mutation		
Elfamycins/kirromycins	*EF-Tu* mutation		
Lipopeptides	*cls* mutation^*^		
	*gdpD* mutation^*^		
Peptide/ansamycins antibiotics	*rpoB*		
Diaminopyrimidines	*dfrE*		
Tetracyclines	*tetM*		
Aminoglycosides	*AAC(6′)-Ii*	*APH(3′)-IIIa*	*AAC(6′)-Ie-APH(2″)-Ia1*
			*AAC(6′)-Ie-APH(2″)-Ia2*
Macrolides/lincosamides/streptogramins		*ermB*	
Glycopeptides		*vanM*	
		*vanHM*	
		*vanYM*	
		*vanSM*	
		*vanRM*	
		*vanA*	
		*vanSA*	
		*vanHA*	
		*vanXA*	
		*vanRA*	
Total	9	12	2

* Genes associated with DAP resistance.

While comparing the genomic sequences of *E. faecium* EF332 and daptomycin-sensitive *E. faecium* DO strain, mutations were found on *cls* and *gdpD* that, respectively, encode cardiolipin synthase and glycerophosphoryl diester phosphodiesterase (Table 4). It was previously reported that mutations in these genes can lead to daptomycin resistance (Humphries et al., 2012; Davlieva et al., 2013; Kelesidis et al., 2013; Lellek et al., 2015; Prater et al., 2019). However, known mutations that confer DAP resistance on these genes were not present in *E. faecium* EF332, leading to the hypothesis that new mutations of the two genes could lead to daptomycin resistance.

**Table 4:** Mutations in *cls* and *gdpD* that are associated with DAP resistance.

Genes	Mutations	References
*cls*	N13I, H215R, R218Q, +MPL110-112	Arias et al., 2011
*cls*	N13T, K59T	Kelesidis et al., 2013
*cls*	H215R, R218Q	Davlieva et al., 2013
*cls*	H215R	Humphries et al., 2012
*cls*	A20D, D27N, R218Q, R267H	Lellek et al., 2015
*cls*	I203V, I269T, S298T	This study
*gdpD**^a^*	H29R	Prater et al., 2019
*gdpD*	P201S	This study

a This mutation was found in DAP-resistant *Enterococcus faecium* but has not been shown to confer DAP resistance.

### New *cls* Mutations Lead to Daptomycin Resistance in *Enterococcus faecium* EF332

In order to find out which mutations on *cls* and/or *gdpD* lead to daptomycin resistance in *E. faecium* EF332 strains, a series of clones were constructed using daptomycin-sensitive *E. faecalis* ATCC 29212 as the parent strain: the EF332-*cls* strain that harbors *cls* from *E. faecium* EF332; the EFDO-*cls* strain that harbors *cls* from *E. faecium* EF332 with identified mutations (V_203_, T_269_, T_298_) reverted to I_203_, I_269_, and S_298_; the EF332-*gdpD* strain that harbors *gdpD* from *E. faecium* EF332; and the EFDO-*gdpD* strain that harbors *gdpD* from *E. faecium* EF332 with identified mutation S_201_ reverted to P_201_; the 29212-pDL278 strain that harbors pDL278 empty vector as control for constructed strains (Table 1). It is shown that the DAP MIC of *E. faecalis* ATCC 29212 increased from 1 to 8 with the introduction of pDL278 vector (Supplementary Table 4). A twofold increase in MIC values for daptomycin was found for EF332-*cls* strain (16 μg/ml) over the EFDO-*cls* strain (8 μg/ml), whereas no change of MIC values for daptomycin was found for EF332-*gdpD* strain (8 μg/ml) over the EFDO-*gdpD* strain (8 μg/ml; Supplementary Table 4). These results suggest that mutations in *cls* can lead to daptomycin resistance, while mutations in *gdpD* cannot.

**Figure 3:**
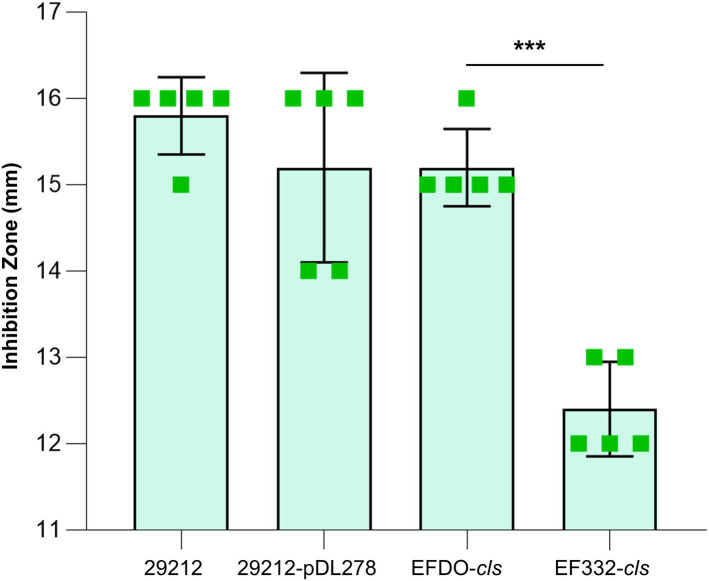
Susceptibility tests of strains based on K-B disk diffusion method. 29212, *Enterococcus faecalis* ATCC 29212; ^***^p<0.001; bar, standard deviation.

**Figure 4:**
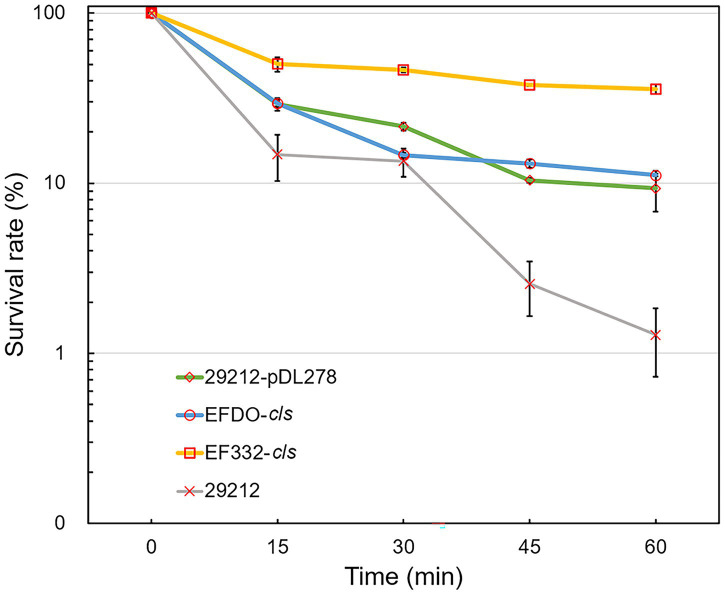
Daptomycin survival assays of *Enterococcus faecalis* strains. 29212, *E. faecalis* ATCC 29212; bar, standard deviation.

**Figure 5:**
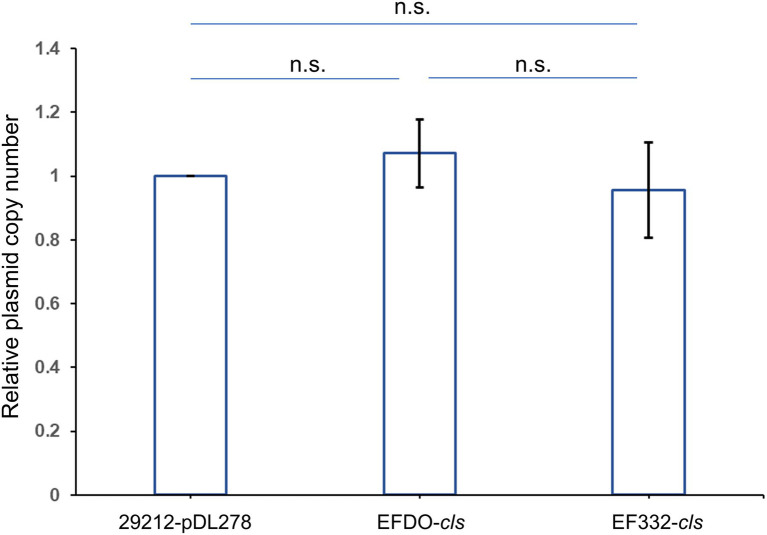
Relative plasmid copy numbers. Relative plasmid copy numbers are defined as relative normalized plasmid copy numbers to 29212-pDL278 strain. Bar, standard deviation; n.s., not significant.

Further susceptibility tests including K-B disk diffusion assays and survival assays support this finding. The inhibition zones of daptomycin with the EF332-*cls* strain are significantly smaller than the EFDO-*cls* strain (*p =* 2.09 × 10^−5^, Figure 3), while no significant difference was found between EFDO-*cls* strain and 29212-pDL278. Similarly, in survival assays, a significantly higher daptomycin resistance strength was found for EF332-*cls* in comparison with EFDO-*cls*, while EFDO-*cls* and 29212-pDL278 respond to daptomycin stress similarly (Figure 4). Additional analysis of plasmid copy numbers confirmed that introducing variants of *cls* did not lead to plasmid copy number fluctuation (Figure 5). It is further interesting to find that in both liquid-media-based analyses, aka the MIC determination and survival analysis, pDL278 introduction increased daptomycin resistance (Figure 4), while in the plate-based K-B disk diffusion assay, pDL278 introduction led to little difference in daptomycin resistance (Figure 3). This prompted us to hypothesize that maybe pDL278 can result in growth status-specific response to daptomycin, but without further evidence we cannot suggest the specific mechanism behind this phenomenon. Despite this difference, in all scenarios and in all assays, introducing *cls* from *E. faecium* EF332 leads to significantly stronger daptomycin resistance than introducing *cls* from *E. faecium* DO, confirming the above suggestion that mutations in *cls* found in *E. faecium* EF332 lead to daptomycin resistance.

### Impact of *cls* and *gdpD* Mutations on Membrane Properties

**Figure 6:**
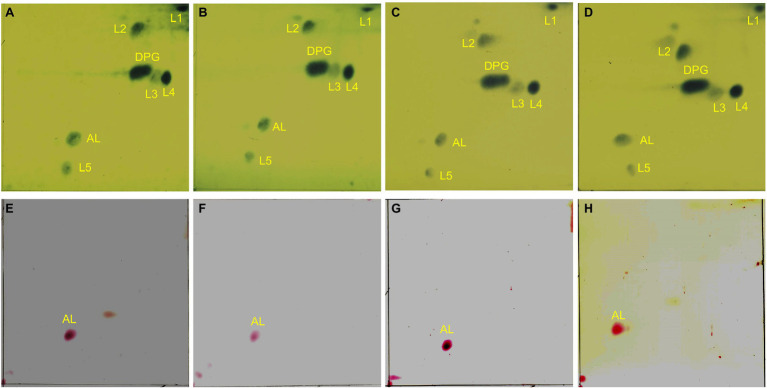
Two-dimensional thin-layer chromatography of phospholipids in *Enterococcus faecium* EF332 strains. Panels **(A–D)** are results of phosphomolybdic acid staining, and all lipids are dyed blue; Panels **(E–H)** are results of ninhydrin staining, and aminolipids are dyed red. Panels **(A,E)** EFDO-*cls*; Panels **(B,F)** EF332-*cls*; Panels **(C,G)** EFDO-*gdpD*; Panels **(D,H)** EF332-*gdpD*. DPG: diphosphatidyl glycerol (cardiolipin); AL, aminolipids; L1–L5, lipids.

**Figure 7:**
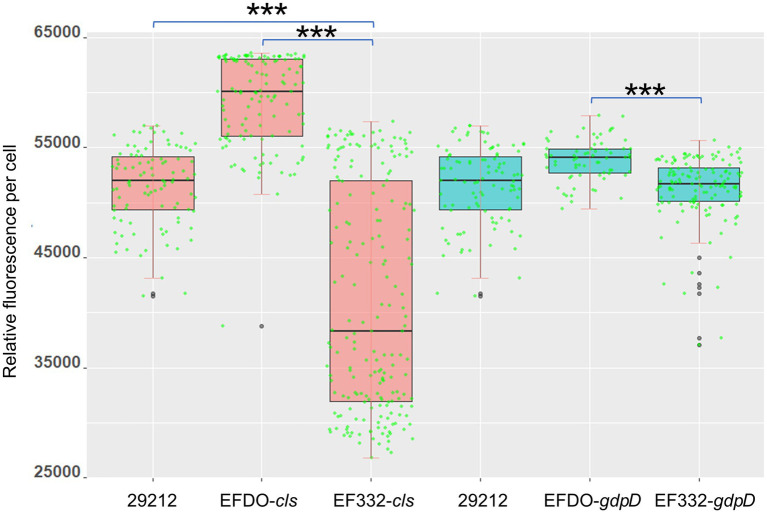
Cell surface charge measured with PLL: FITC fluorescence. 29212, *Enterococcus faecalis* ATCC 29212, ^***^p <0.001.

Both *cls* and *gdpD* genes are related to phospholipid metabolism in CM: The *cls* gene encodes cardiolipin synthase, and *gdpD* gene encodes glycerophosphoryl diester phosphodiesterase (Arias et al., 2011). We therefore hypothesized that mutations in these two genes alter membrane properties, which in turn leads to alteration in membrane–daptomycin interactions. To confirm this, two-dimensional thin-layer chromatography (2D-TLC) was performed to analyze the changes of membrane lipids components caused by mutations. As shown in Figure 6, mutation in *gdpD* altered the aminolipid composition of CM, while mutations in *cls* did not significantly alter the lipid composition of CM. This finding suggests a putative role of *gdpD* in the biosynthesis of relatively poorly characterized aminolipid. Furthermore, membrane surface charges were quantified using positively charged fluorescence dye poly-_L_-lysine conjugated to fluorescein isothiocyanate (PLL: FITC). A significant reduction of binding to PLL: FITC was found between EF332-*cls* and *E. faecalis* ATCC 29212 (*p* = 6.27 × 10^−65^), as well as between EF332-*cls* and EFDO-*cls* (*p* = 2.20 × 10^−101^; Figure 7). This significant change suggests that a reduction of membrane negative charges followed *cls* mutations, which can lead to weakened binding of positively charged DAP-Ca^2+^ to the membrane, the target for the bactericidal effect of daptomycin. This finding suggests that *cls* mutations lead to daptomycin resistance by reduction of membrane negative charges. Only minor changes of membrane negative potentials were found for *gdpD* mutation strain (Figure 7), in consistent with the finding that *gdpD* mutation did not lead to increase of daptomycin resistance.

**Figure 8:**
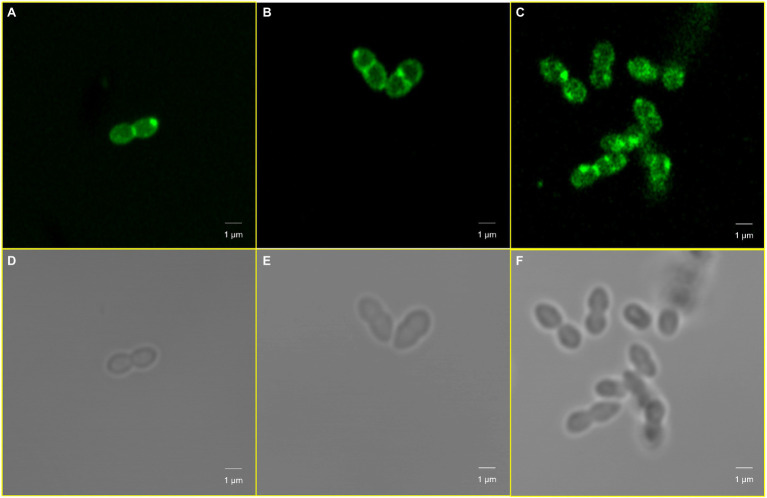
Cardiolipin redistribution caused by *cls* mutations. Panels **(A,D)** *Enterococcus faecalis* ATCC 29212; Panels **(B,E)** EFDO-*cls*; Panels **(C,F)** EF332-*cls.*

We further sought to investigate whether *cls* mutations also alter the distribution of cardiolipin produced by *cls*-encoded cardiolipin synthase. A specific cardiolipin binding fluorescent probe, 10-*N*-nonyl acridine orange (NAO), was used to observe the distribution of cardiolipins in CM. A redistribution of cardiolipin in CM was found (Figure 8). In *E. faecalis* ATCC 29212 (Figures 8A,D) and EFDO-*cls* (Figures 8B,E), the cardiolipin-rich regions were mainly located at the cell septum or at both poles, while in the *cls*-mutant strain EF332-*cls*, the cardiolipin-rich regions were scattered all over the cell, leading to a more even distribution and reduced abundance at septa (Figures 8C,F). This is in agreement with previous suggestion that daptomycin resistance may be resulted from redistribution of phospholipids and diverting DAP from effective targets at the septum (Heidary et al., 2018).

### Structure Predictions Suggest That Cls Mutations May Affect Phospholipase Activity

**Figure 9:**
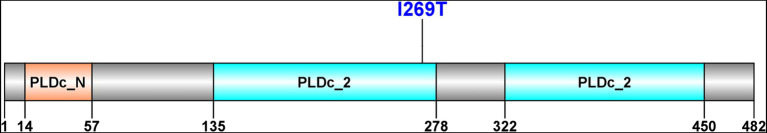
Domain structure prediction of Cls with Pfam.

**Figure 10:**
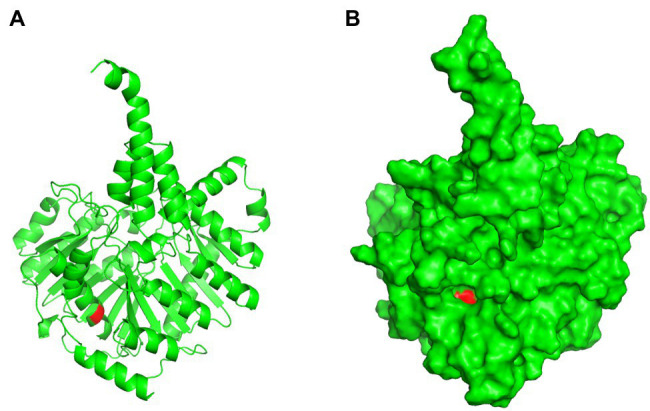
Three-dimensional structure prediction of Cls I269T mutant. Panel **(A)**, cartoon presentation; Panel **(B)**, surface presentation. Red color indicates T269 residue.

Pfam was used to predict Cls domain structures, leading to the finding that the identified I269T mutation in this work is located in one of phospholipase D-like domains (Figure 9). As a three-dimensional structure of Cls is not available, we predicted its three-dimensional structure by AlphaFold 2 (Figure 10). Interestingly, T269 of the Cls mutant is located at the bottom of a pocket that could potentially bind substrate for catalysis (Figure 10B). This is in agreement with domain structure binding, which leads to the suggestion that mutations of Cls may affect the phospholipase activity of this cardiolipin synthase.

### Mutations in *cls* Did Not Lead to Changes in Cell Ultrastructure

**Figure 11:**
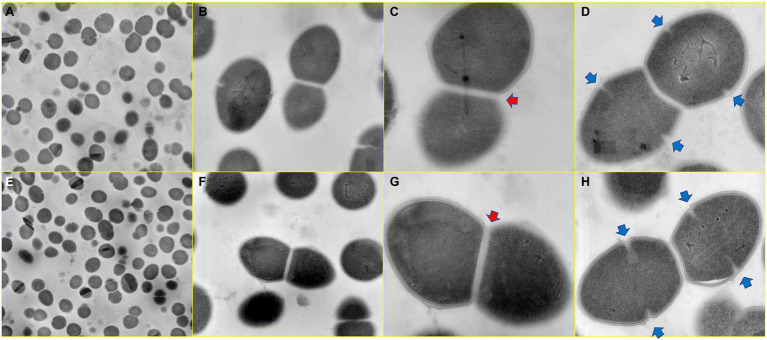
Ultrastructure of *cls* parent and mutant strains. Panels **(A–D)** *Enterococcus faecalis* ATCC 29212; Panels **(E–H)** EF332-*cls*. Image magnifications for Panels **(A,E)**, Panels **(B,F)**, Panels **(C,G)**, and Panels **(D,H)** are 5,000, 20,000, 50,000, and 50,000, respectively. The red arrow marks a single-septum structure, and the blue arrow marks multiple-septum structures.

Using transmission electron microscopy, we were able to compare whether *cls* mutations caused changes in cell envelope and septa between *cls* mutant and parent strains. As shown in Figure 11, for parent strain *E. faecalis* ATCC 29212 and mutant strain EF332-*cls*, the cell surfaces and septum of both strains were smooth, and both single-septum (red arrows) and multiple-septum morphology (blue arrows) were observed and their morphology were similar. This result suggests that *cls* mutations does not change the structure of cell envelope.

## Discussion

As VRE has spread extensively and treatment options have become increasingly limited, DAP is considered to be one of last resort antibiotics for VRE. Assessing DAP resistance, particularly in a vancomycin-resistant strain, is therefore of particular interest. In this work, a DAP-resistant VRE (*E. faecium* EF332) was identified and investigated to understand its mechanism for DAP resistance. Based on whole-genome sequencing and genome comparison, new mutations of two genes associated with phospholipid metabolism, *cls* and *gdpD*, were identified and suspected to be involved in DAP resistance. Mutations in the two genes were shown to be necessary for DAP resistance in previous studies (Arias et al., 2011). Therefore, investigations of the identified new mutations were carried out to verify their relationship with DAP resistance.

Results of this work suggest that mutations in *cls* alone can confer DAP resistance. The c*ls* gene encodes a cardiolipin synthase, which catalyzes the production of cardiolipin from two molecules of phosphatidylglycerol. Previous studies have suggested that an increase in the concentration of cardiolipin in CM can divert more DAP from its target septum to other sites, thereby improving DAP resistance (Zhang et al., 2014; Tran et al., 2015). Some bacteria can change the membrane anionic microdomain to resist DAP by reducing the negatively charged membrane phospholipid composition (Mishra et al., 2017). While analysis of phospholipid composition by 2D-TLC showed that the *cls* mutations did not change the phospholipid composition of CM (including cardiolipin levels; Figure 6), the *cls* mutations led to decrease in negative charges on the cell surface (Figure 7) and may lead to a reduced adhesion to DAP. It has been reported that membrane lipids are asymmetrically distributed in the inner and outer leaflet of CM (Tannert et al., 2003). We therefore postulated that *cls* mutations do not change the level of cardiolipin synthesized but change the distribution of cardiolipin in CM, leading to reduction of cardiolipin in the outer leaflet of CM, thereby reducing membrane surface negative charges and the adhesion of DAP (Mukhopadhyay et al., 2007). It was previously suggested that DAP resistance can also be caused by redistribution of anionic phospholipids and diverting from effective targets at the septum (Tran et al., 2013b; Heidary et al., 2018). In this work, mutations of *cls* led to a more evenly distributed cardiolipins and reduction in septa (Figure 8C), in agreement with this theory. Therefore, we suspect the resistance to DAP found in this work is also attributable to the redistribution of cardiolipin away from the cell septa. Both events, reduction of cellular negative charge and redistribution of phospholipids, may work together or even synergistically to complete the development of DAP resistance in *E. faecium*. We also found that *cls* mutations did not lead to changes in cell ultrastructure, which is inconsistent with the previously reported results observed in S613 (DAP susceptible) and R712 (DAP-resistant, deletion of Lys at position 61) strains (Arias et al., 2011). This may be due to different effects of different mutated sites on the cell envelope and septum.

Out of the three mutations in *cls*, both I203V and S298T are mutations to similar amino acids, while I269T is a mutation from hydrophobic residue to polar residue. We therefore speculate that I269T is the effective mutation conferring DAP resistance. This is further supported by predictions of domain structure (Figure 9) and three-dimensional structure (Figure 10), which suggests that I269T mutation may alter the catalytic activity of cardiolipin synthase. Another DAP-resistant strain of *E. faecium* found by our research team also showed the same I269T mutation (data not shown), which also supports our prediction.

Although the MIC of DAP on *E. faecium* EF332 is not high (8 μg/ml), it has a high potential transmission risk. MLST analysis revealed that the sequence type of *E. faecium* EF332 is ST192, belonging to epidemic hospital strains (clade A1), which included polyclonal complex 17 (CC17; Lebreton et al., 2013, 2018). *Enterococcus faecium* of CC17 is high-risk clonal lineages, which is often associated with nosocomial VRE outbreak and leads to severe morbidity and mortality (Lee et al., 2019). Worryingly, plasmid pEF332-2 in this study carried two high-risk gene clusters *vanA* and *vanM*. *VanA* gene clusters were also found on genomic islands GIs011 and prophage (Figure 1). This means that vancomycin resistance of this strain could easily disseminate, leading to higher levels of prevalence. One more thing to note is *vanM* gene has only been previously reported in Shanghai, Chengdu, Hangzhou, and Beijing in China (Chen et al., 2014, 2015; Sun et al., 2019a,b). *Enterococcus faecium* EF332 carrying *vanM* genes in this study was found in Jinan of China, suggesting that *vanM* gene may have already spread nationwide.

## Conclusion

In this study, a clinical DAP-resistant VRE was identified. Whole-genome sequencing revealed the genetic background for multidrug resistance. A new plasmid pEF332-2 was found to carry two vancomycin resistance clusters, a rare phenomenon reported only twice so far. The genetic basis for daptomycin was investigated, suggesting new mutations in *cls* gene confer DAP resistance. The mechanisms of DAP resistance were further investigated, showing that *cls* mutations lead to significant decrease of membrane surface negative charges and the redistribution of cardiolipin in CM, both of which contribute to DAP resistance. This work reports the genetic basis of multidrug resistance of a daptomycin- and vancomycin-resistant *E. faecium*, and new mutations of *cls* that leads to resistance of daptomycin, a key last-resort antibiotic.

## Data Availability Statement

To fully understand the genetic basis of multidrug resistance in E. faecium EF332, whole-genome sequences were obtained with third-generation PacBio sequencing accompanied by second-generation Illumina sequencing (The sequencing data were deposited in GenBank with accession numbers CP058891-CP058895).

## Author Contributions

HX and MW designed this study and revised the manuscript. XZ was responsible for sample collection and strain identification and participated in experimental design. HS helped to analyze membrane lipid compositions. JH, LL, MZ, QC, and YM participated in the experiments and assisted in collecting experimental data. WL performed experiments, collected and analyzed the data, and wrote the draft of the manuscript. All authors contributed to the article and approved the submitted version.

## Funding

This work was supported by the National Key Research and Development Program of China (grant number 2021YFE0199800), Key R&D Program of Shandong Province (grant number 2020CXGC011305); the National Natural Science Foundation of China (Grant numbers 31770042 and 31770043); and Shandong Provincial Natural Science Foundation (grant number ZR2020MH308).

## Conflict of Interest

The authors declare that the research was conducted in the absence of any commercial or financial relationships that could be construed as a potential conflict of interest.

## Publisher’s Note

All claims expressed in this article are solely those of the authors and do not necessarily represent those of their affiliated organizations, or those of the publisher, the editors and the reviewers. Any product that may be evaluated in this article, or claim that may be made by its manufacturer, is not guaranteed or endorsed by the publisher.

## Footnotes

^1^ https://cge.food.dtu.dk/services/MLST/

^2^ https://cge.food.dtu.dk/services/PlasmidFinder/

## References

[ref1] Ardui S. Ameur A. Vermeesch J. R. Hestand M. S. (2018). Single molecule real-time (SMRT) sequencing comes of age: applications and utilities for medical diagnostics. Nucleic Acids Res. 46, 2159–2168. doi: 10.1093/nar/gky066, PMID: 29401301 PMC5861413

[ref2] Arias C. A. Murray B. E. (2012). The rise of the *Enterococcus*: beyond vancomycin resistance. Nat. Rev. Microbiol. 10, 266–278. doi: 10.1038/nrmicro2761, PMID: 22421879 PMC3621121

[ref3] Arias C. A. Panesso D. McGrath D. M. Qin X. Mojica M. F. Miller C.. (2011). Genetic basis for *in vivo* daptomycin resistance in enterococci. N. Engl. J. Med. 365, 892–900. doi: 10.1056/NEJMoa1011138.Genetic, PMID: 21899450 PMC3205971

[ref4] Arndt D. Grant J. R. Marcu A. Sajed T. Pon A. Liang Y.. (2016). PHASTER: a better, faster version of the PHAST phage search tool. Nucleic Acids Res. 44, W16–W21. doi: 10.1093/nar/gkw387, PMID: 27141966 PMC4987931

[ref5] Ashburner M. Ball C. A. Blake J. A. Botstein D. Butler H. Cherry J. M.. (2000). Gene ontology: tool for the unification of biology. Nat. Genet. 25, 25–29. doi: 10.1038/75556, PMID: 10802651 PMC3037419

[ref6] Bairoch A. Apweiler R. (2000). The SWISS-PROT protein sequence database and its supplement TrEMBL in 2000. Nucleic Acids Res. 28, 45–48. doi: 10.1128/AAC.02435-16, PMID: 10592178 PMC102476

[ref7] Bender J. K. Cattoir V. Hegstad K. Sadowy E. Coque T. M. Westh H.. (2018). Update on prevalence and mechanisms of resistance to linezolid, tigecycline and daptomycin in enterococci in Europe: towards a common nomenclature. Drug Resist. Updat. 40, 25–39. doi: 10.1016/j.drup.2018.10.002, PMID: 30447411

[ref8] Besemer J. Lomsadze A. Borodovsky M. (2001). GeneMarkS: a self-training method for prediction of gene starts in microbial genomes. Implications for finding sequence motifs in regulatory regions. Nucleic Acids Res. 29, 2607–2618. doi: 10.1093/nar/29.12.2607, PMID: 11410670 PMC55746

[ref9] Carver P. L. Whang E. VandenBussche H. L. Kauffman C. A. Malani P. N. (2003). Risk factors for arthralgias or myalgias associated with quinupristin-dalfopristin therapy. Pharmacotherapy 23, 159–164. doi: 10.1592/phco.23.2.159.32078 12587804

[ref10] Cattoir V. Giard J. C. (2014). Antibiotic resistance in *Enterococcus faecium* clinical isolates. Expert Rev. Anti-Infect. Ther. 12, 239–248. doi: 10.1586/14787210.2014.870886 24392717

[ref11] Chen C. Sun J. Guo Y. Lin D. Guo Q. Hu F.. (2015). High prevalence of *vanM* in vancomycin-resistant *Enterococcus faecium* isolates from Shanghai, China. Antimicrob. Agents Chemother. 59, 7795–7798. doi: 10.1128/AAC.01732-15, PMID: 26369966 PMC4649207

[ref12] Chen C. Xu X. Qu T. Yu Y. Ying C. Liu Q.. (2014). Prevalence of the fosfomycin-resistance determinant, *fosB3*, in *Enterococcus faecium* clinical isolates from China. J. Med. Microbiol. 63, 1484–1489. doi: 10.1099/jmm.0.077701-0, PMID: 25102907

[ref13] Chow A. Win N. N. Ng P. Y. Lee W. Win M. K. (2016). Vancomycin-resistant enterococci with reduced daptomycin susceptibility in Singapore: prevalence and associated factors. Epidemiol. Infect. 144, 2540–2545. doi: 10.1017/S0950268816000923, PMID: 27174845 PMC9150451

[ref14] CLSI (2018). Performance standards for antimicrobial susceptibility testing. Clin. Lab. Stand. Inst. 1–296.

[ref15] Davlieva M. Zhang W. Arias C. A. Shamoo Y. (2013). Biochemical characterization of cardiolipin synthase mutations associated with daptomycin resistance in enterococci. Antimicrob. Agents Chemother. 57, 289–296. doi: 10.1128/AAC.01743-12, PMID: 23114777 PMC3535954

[ref16] Depardieu F. Podglajen I. Leclercq R. Collatz E. Courvalin P. (2007). Modes and modulations of antibiotic resistance gene expression. Clin. Microbiol. Rev. 20, 79–114. doi: 10.1128/CMR.00015-06, PMID: 17223624 PMC1797629

[ref17] EUCAST (2017). The European committee on antimicrobial susceptibility testing. Eur. Comm. Antimicrob. Susceptibility Test 1–146.

[ref18] Fischer A. Yang S. J. Bayer A. S. Vaezzadeh A. R. Herzig S. Stenz L.. (2011). Daptomycin resistance mechanisms in clinically derived *Staphylococcus aureus* strains assessed by a combined transcriptomics and proteomics approach. J. Antimicrob. Chemother. 66, 1696–1711. doi: 10.1093/jac/dkr195, PMID: 21622973 PMC3133485

[ref19] Freitas A. R. Tedim A. P. Novais C. Ruiz-Garbajosa P. Werner G. Laverde-Gomez J. A.. (2010). Global spread of the hylEfm colonization-virulence gene in megaplasmids of the *Enterococcus faecium* CC17 polyclonal subcluster. Antimicrob. Agents Chemother. 54, 2660–2665. doi: 10.1128/AAC.00134-10, PMID: 20385861 PMC2876360

[ref20] Galperin M. Y. Makarova K. S. Wolf Y. I. Koonin E. V. (2014). Expanded microbial genome coverage and improved protein family annotation in the COG database. Nucleic Acids Res. 43, D261–D269. doi: 10.1093/nar/gku1223, PMID: 25428365 PMC4383993

[ref21] Gonzalez-Ruiz A. Seaton R. A. Hamed K. (2016). Daptomycin: an evidence-based review of its role in the treatment of gram-positive infections. Infect. Drug Resist. 9, 47–58. doi: 10.2147/IDR.S99046, PMID: 27143941 PMC4846043

[ref22] Grein F. Müller A. Scherer K. M. Liu X. Ludwig K. C. Klöckner A.. (2020). Ca^2+^-Daptomycin targets cell wall biosynthesis by forming a tripartite complex with undecaprenyl-coupled intermediates and membrane lipids. Nat. Commun. 11:1455. doi: 10.1038/s41467-020-15257-1, PMID: 32193379 PMC7081307

[ref23] Hashimoto Y. Taniguchi M. Uesaka K. Nomura T. Hirakawa H. Tanimoto K.. (2019). Novel multidrug-resistant enterococcal mobile linear plasmid pELF1 encoding *vanA* and *vanM* gene clusters from a Japanese vancomycin-resistant enterococci isolate. Front. Microbiol. 10:2568. doi: 10.3389/fmicb.2019.02568, PMID: 31798546 PMC6863802

[ref24] Heidary M. Khosravi A. D. Khoshnood S. Nasiri M. J. Soleimani S. Goudarzi M. (2018). Daptomycin. J. Antimicrob. Chemother. 73, 1–11. doi: 10.1093/jac/dkx349 29059358

[ref25] Herc E. S. Kauffman C. A. Marini B. L. Perissinotti A. J. Miceli M. H. (2017). Daptomycin nonsusceptible vancomycin resistant *Enterococcus* bloodstream infections in patients with hematological malignancies: risk factors and outcomes. Leuk. Lymphoma 58, 2852–2858. doi: 10.1080/10428194.2017.1312665, PMID: 28402152

[ref26] Ho S. N. Hunt H. D. Horton R. M. Pullen J. K. Pease L. R. (1989). Site-directed mutagenesis by overlap extension using the polymerase chain reaction. Gene 77, 51–59. doi: 10.1016/0378-1119(89)90358-2 2744487

[ref27] Hogan H. L. Hachem R. Y. Neuhauser M. Raad I. I. Coyle E. (2010). Clinical experience of linezolid in bone marrow transplantation patients. J. Pharm. Pract. 23, 352–357. doi: 10.1177/0897190009358773, PMID: 21507835

[ref28] Hsiao W. Wan I. Jones S. J. Brinkman F. S. L. (2003). IslandPath: aiding detection of genomic islands in prokaryotes. Bioinformatics 19, 418–420. doi: 10.1093/bioinformatics/btg004, PMID: 12584130

[ref29] Humphries R. M. Kelesidis T. Tewhey R. Rose W. E. Schork N. Nizet V.. (2012). Genotypic and phenotypic evaluation of the evolution of high-level daptomycin nonsusceptibility in vancomycin-resistant *Enterococcus faecium*. Antimicrob. Agents Chemother. 56, 6051–6053. doi: 10.1128/AAC.01318-12, PMID: 22948885 PMC3486580

[ref30] Hussain K. Ullah S. Tahir H. Alkilani W. Z. Naeem M. Vinod N. R.. (2016). Daptomycin- vancomycin-resistant *Enterococcus faecium* native valve endocarditis: successfully treated with off-label quinupristin-dalfopristin. J. Investig. Med. High Impact Case Rep. 4, 3–5. doi: 10.1177/2324709616665408, PMID: 27689100 PMC5027907

[ref31] Jia B. Raphenya A. R. Alcock B. Waglechner N. Guo P. Tsang K. K.. (2016). CARD 2017: expansion and model-centric curation of the comprehensive antibiotic resistance database. Nucleic Acids Res. 45, D566–D573. doi: 10.1093/nar/gkw1004, PMID: 27789705 PMC5210516

[ref32] Jumper J. Evans R. Pritzel A. Green T. Figurnov M. Ronneberger O.. (2021). Highly accurate protein structure prediction with AlphaFold. Nature 596, 583–589. doi: 10.1038/s41586-021-03819-2, PMID: 34265844 PMC8371605

[ref33] Kelesidis T. Tewhey R. Humphries R. M. (2013). Evolution of high-level daptomycin resistance in *Enterococcus faecium* during daptomycin therapy is associated with limited mutations in the bacterial genome. J. Antimicrob. Chemother. 68, 1926–1928. doi: 10.1093/jac/dkt117, PMID: 23580562 PMC6373497

[ref34] Komagata K. Suzuki K. I. (1988). 4 lipid and cell-wall analysis in bacterial systematics. Methods Microbiol. 19, 161–207. doi: 10.1016/S0580-9517(08)70410-0

[ref35] LaPlante K. L. Rybak M. J. (2004). Daptomycin – a novel antibiotic against gram-positive pathogens. Expert. Opin. Pharmacother. 5, 2321–2331. doi: 10.1517/14656566.5.11.2321 15500379

[ref36] Lebreton F. Valentino M. D. Schaufler K. Earl A. M. Cattoir V. Gilmore M. S. (2018). Transferable vancomycin resistance in clade B commensal-type *Enterococcus faecium*. J. Antimicrob. Chemother. 73, 1479–1486. doi: 10.1093/jac/dky039, PMID: 29462403 PMC5961315

[ref37] Lebreton F. van Schaik W. Manson McGuire A. Godfrey P. Griggs A. Mazumdar V.. (2013). Emergence of epidemic multidrug-resistant *Enterococcus faecium* from animal and commensal strains. MBio 4:00534-13. doi: 10.1128/mBio.00534-13, PMID: 23963180 PMC3747589

[ref38] Lee T. Pang S. Abraham S. Coombs G. W. (2019). Antimicrobial-resistant CC17 *Enterococcus faecium*: the past, the present and the future. J. Glob. Antimicrob. Resist. 16, 36–47. doi: 10.1016/j.jgar.2018.08.016, PMID: 30149193

[ref39] Lellek H. Franke G. C. Ruckert C. Wolters M. Wolschke C. Christner M.. (2015). Emergence of daptomycin non-susceptibility in colonizing vancomycin-resistant *Enterococcus faecium* isolates during daptomycin therapy. Int. J. Med. Microbiol. 305, 902–909. doi: 10.1016/j.ijmm.2015.09.005, PMID: 26454536

[ref40] Li W. Jaroszewski L. Godzik A. (2002). Tolerating some redundancy significantly speeds up clustering of large protein databases. Bioinformatics 18, 77–82. doi: 10.1093/bioinformatics/18.1.77, PMID: 11836214

[ref41] Matsumoto K. Takeshita A. Ikawa K. Shigemi A. Yaji K. Shimodozono Y.. (2010). Higher linezolid exposure and higher frequency of thrombocytopenia in patients with renal dysfunction. Int. J. Antimicrob. Agents 36, 179–181. doi: 10.1016/j.ijantimicag.2010.02.019, PMID: 20392606

[ref42] Mileykovskaya E. Dowhan W. Birke R. L. Zheng D. Lutterodt L. Haines T. H. (2001). Cardiolipin binds nonyl acridine orange by aggregating the dye at exposed hydrophobic domains on bilayer surfaces. FEBS Lett. 507, 187–190. doi: 10.1016/S0014-5793(01)02948-9, PMID: 11684095

[ref43] Mishra N. N. Tran T. T. Seepersaud R. Garcia-De-La-Maria C. Faull K. Yoon A.. (2017). Perturbations of phosphatidate cytidylyltransferase (CdsA) mediate daptomycin resistance in *Streptococcus mitis/oralis* by a novel mechanism. Antimicrob. Agents Chemother. 61, 1–13. doi: 10.1128/AAC.02435-16, PMID: 28115347 PMC5365703

[ref44] Mistry J. Chuguransky S. Williams L. Qureshi M. Salazar G. A. Sonnhammer E. L. L.. (2021). Pfam: The protein families database in 2021. Nucleic Acids Res. 49, D412–D419. doi: 10.1093/nar/gkaa913, PMID: 33125078 PMC7779014

[ref45] Mukhopadhyay K. Whitmire W. Xiong Y. O. Molden J. Jones T. Peschel A.. (2007). *In vitro* susceptibility of *Staphylococcus aureus* to thrombin-induced platelet microbicidal protein-1 (tPMP-1) is influenced by cell membrene phospholipid composition and asymmetry. Microbiology 153, 1187–1197. doi: 10.1099/mic.0.2006/003111-0, PMID: 17379728

[ref46] Munoz-Price L. S. Lolans K. Quinn J. P. (2005). Emergence of resistance to daptomycin during treatment of vancomycin-resistant *Enterococcus faecalis* infection. Clin. Infect. Dis. 41, 565–566. doi: 10.1086/432121, PMID: 16028170

[ref47] Prater A. G. Mehta H. H. Beabout K. Supandy A. Miller W. R. Tran T. T.. (2021). Daptomycin resistance in *Enterococcus faecium* can be delayed by disruption of the LiaFSR stress response pathway. Antimicrob. Agents Chemother. 65, 1–6. doi: 10.1128/AAC.01317-20, PMID: 33468468 PMC8097453

[ref48] Prater A. G. Mehta H. H. Kosgei A. J. Miller W. R. Tran T. T. Arias C. A.. (2019). Environment shapes the accessible daptomycin resistance mechanisms in *Enterococcus faecium*. Antimicrob. Agents Chemother. 63, 3–5. doi: 10.1128/AAC.00790-19, PMID: 31332078 PMC6761497

[ref49] Punta M. Coggill P. C. Eberhardt R. Y. Mistry J. Tate J. Boursnell C.. (2011). The Pfam protein families database. Nucleic Acids Res. 32, 138D–1141D. doi: 10.1093/nar/gkh121, PMID: 14681378 PMC308855

[ref50] Qian Y. Zhao X. (2014). A comparison of methods for the extraction of bacterial genomic DNA in rat feces. Sci. Technol. Food Ind. 35, 166–169. doi: 10.13386/j.issn1002-0306.2014.04.005

[ref51] Ren J. Wen L. Gao X. Jin C. Xue Y. Yao X. (2009). DOG 1.0: illustrator of protein domain structures. Cell Res. 19, 271–273. doi: 10.1038/cr.2009.6, PMID: 19153597

[ref52] Saier M. H. Jr. Reddy V. S. Tamang D. G. Västermark Å. (2013). The transporter classification database. Nucleic Acids Res. 42, D251–D258. doi: 10.1093/nar/gkt1097, PMID: 24225317 PMC3964967

[ref53] Schwarz F. V. Perreten V. Teuber M. (2001). Sequence of the 50-kb conjugative multiresistance plasmid pRE25 from *Enterococcus faecalis* RE25. Plasmid 46, 170–187. doi: 10.1006/plas.2001.1544, PMID: 11735367

[ref54] Sletvold H. Johnsen P. J. Simonsen G. S. Aasnæs B. Sundsfjord A. Nielsen K. M. (2007). Comparative DNA analysis of two *vanA* plasmids from *Enterococcus faecium* strains isolated from poultry and a poultry farmer in Norway. Antimicrob. Agents Chemother. 51, 736–739. doi: 10.1128/AAC.00557-06, PMID: 17116680 PMC1797720

[ref55] Suleyman G. Mahan M. Zervos M. J. (2017). Comparison of daptomycin and linezolid in the treatment of vancomycin-resistant *Enterococcus faecium* in the absence of endocarditis. Infect. Dis. Clin. Pract. 25, 151–154. doi: 10.1097/IPC.0000000000000482

[ref56] Sun H. L. Liu C. Zhang J. J. Zhou Y. M. Xu Y. C. (2019a). Molecular characterization of vancomycin-resistant enterococci isolated from a hospital in Beijing, China. J. Microbiol. Immunol. Infect. 52, 433–442. doi: 10.1016/j.jmii.2018.12.008, PMID: 30827858

[ref57] Sun L. Qu T. Wang D. Chen Y. Fu Y. Yang Q.. (2019b). Characterization of *vanM* carrying clinical *Enterococcus* isolates and diversity of the suppressed *vanM* gene cluster. Infect. Genet. Evol. 68, 145–152. doi: 10.1016/j.meegid.2018.12.015, PMID: 30553064

[ref58] Sun L. Xu J. Wang W. He F. (2020). Emergence of *vanA*-type vancomycin-resistant *Enterococcus faecium* ST 78 strain with a rep2-type plasmid carrying a Tn1546-like element isolated from a urinary tract infection in China. Infect. Drug Resist. 13, 949–955. doi: 10.2147/IDR.S247569, PMID: 32308438 PMC7135120

[ref59] Tannert A. Pohl A. Pomorski T. Herrmann A. (2003). Protein-mediated transbilayer movement of lipids in eukaryotes and prokaryotes: the relevance of ABC transporters. Int. J. Antimicrob. Agents 22, 177–187. doi: 10.1016/S0924-8579(03)00217-6, PMID: 13678819

[ref60] Tran T. T. Munita J. M. Arias C. A. (2015). Mechanisms of drug resistance: daptomycin resistance. Ann. N. Y. Acad. Sci. 1354, 32–53. doi: 10.1111/nyas.12948, PMID: 26495887 PMC4966536

[ref61] Tran T. T. Panesso D. Gao H. Roh J. H. Munita J. M. Reyes J.. (2013a). Whole-genome analysis of a daptomycin-susceptible *Enterococcus faecium* strain and its daptomycin-resistant variant arising during therapy. Antimicrob. Agents Chemother. 57, 261–268. doi: 10.1128/AAC.01454-12, PMID: 23114757 PMC3535923

[ref62] Tran T. T. Panesso D. Mishra N. N. Mileykovskaya E. Guan Z. Munita J. M.. (2013b). Daptomycin-resistant *Enterococcus faecalis* diverts the antibiotic molecule from the division septum and remodels cell membrane phospholipids. MBio 4:e00281-13. doi: 10.1128/mBio.00281-13, PMID: 23882013 PMC3735187

[ref63] Uttley A. C. Collins C. H. Naidoo J. George R. C. (1988). Vancomycin-resistant enterococci. Lancet 331, 57–58. doi: 10.1016/S0140-6736(88)91037-9 2891921

[ref64] Vading M. Samuelsen H. B. Sundsfjord A. S. Giske C. G. (2011). Comparison of disk diffusion, Etest and VITEK2 for detection of carbapenemase-producing *Klebsiella pneumoniae* with the EUCAST and CLSI breakpoint systems. Clin. Microbiol. Infect. 17, 668–674. doi: 10.1111/j.1469-0691.2010.03299.x, PMID: 20649801

[ref65] Zhang T. H. Muraih J. K. Tishbi N. Herskowitz J. Victor R. L. Silverman J.. (2014). Cardiolipin prevents membrane translocation and permeabilization by daptomycin. J. Biol. Chem. 289, 11584–11591. doi: 10.1074/jbc.M114.554444, PMID: 24616102 PMC4002069

[ref66] Zhou Y. Liang Y. Lynch K. H. Dennis J. J. Wishart D. S. (2011). PHAST: A fast phage search tool. Nucleic Acids Res. 39, W347–W352. doi: 10.1093/nar/gkr485, PMID: 21672955 PMC3125810

