# Supplementary material for: New Mutations in cls Lead to Daptomycin Resistance in a Clinical Vancomycin- and Daptomycin-Resistant Enterococcus faecium Strain
Source: Front Microbiol. 2022 Jun 21;13:896916. doi: 10.3389/fmicb.2022.896916 (PMC9253605; doi:10.3389/fmicb.2022.896916)
Supplement: Supplementary file 3 [file Data_Sheet_1.docx]

Supplementary Material

**Supplementary Table 1**. **Primers used in this study.** a, protective bases plus BamHI sites; b, protective bases plus SalI sites.

| **Gene fragment** | **Primer name** | **Primer sequences** | **Product size (bp)** | **Notes** |
| --- | --- | --- | --- | --- |
| *gdpD* | gdpdF | (CGGGATCCC)^a^AAACCACTGTCGATTTTCTTATATAGTATGGC | 2100 | In *E. faecium* EF332 |
|  | gdpdR | (ACGCGTCGAC)^b^TTAAGCACACATTATGAGCGTCAAAC |  |  |
| *cls* | clsF | (CGGGATCC)^a^CCAAAGTTAATTTCGCTCATGCGAG | 1776 | In *E. faecium* EF332 |
|  | clsR | (ACGCGTCGAC)^b^TTCATTGGTTCACTTGGCTCGAT |  |  |
| Site mutated *gdpD* | hfgdpd1 | (CGGGATCCC)^a^AAACCACTGTCGATTTTCTTATATAGTATGGC | 814 | SOE PCR |
|  | hfgdpd2 | TGGCTGCTCGAAAAGGACGATCTCGTAGAATCAT |  |  |
|  | hfgdpd3 | TCGTCCTTTTCGAGCAGCCATTCGGG | 1306 |  |
|  | hfgdpd4 | (ACGCGTCGAC)^b^TTAAGCACACATTATGAGCGTCAAAC |  |  |
| Site mutated *cls* | hfcls1 | (CGGGATCCC)^a^CCAAAGTTAATTTCGCTCATGCGAG | 688 | SOE PCR |
|  | hfcls2 | CCATTCACTCTACTGGTGATATAGCTGTTCAAATCGTGACAAGC |  |  |
|  | hfcls3 | TTTGAACAGCTATATCACCAGTAGAGTGAATGGACGGA | 134 |  |
|  | hfcls4 | TATTTACGGAGAAGCGGTGTATATCTTGCAAAACCGTTTTTTGATGG |  |  |
|  | hfcls5 | TTGCAAGATATACACCGCTTCTCCGTAAATACGCAAATG | 220 |  |
|  | hfcls6 | AGCAGGCGGAGAAATCGCATTTTTCTTTCCTCTATTTGTTCC |  |  |
|  | hfcls7 | TGCGATTTCTCCGCCTGCTTTTTTCAAATTCTGAA | 817 |  |
|  | hfcls8 | (ACGCGTCGAC)^b^TTCATTGGTTCACTTGGCTCGAT |  |  |
| 16S rDNA | q16sF | CCCAGATGGGATTAGCTTGT | 106 | For qPCR, (Kim and Lee, 2014) |
|  | q16sR | TCTGGACCGTGTCTCAGTTC |  |  |
| pDL278 | qpdlF | CGAGGTATGTAGGCGGTGCTA | 148 | For qPCR |
|  | qpdlR | GGTGGTTTGTTTGCCGGATCAA |  |  |
|  | m13F | GTAAAACGACGGCCAGT | 103-2100 | For verification |
|  | m13R | CAGGAAACAGCTATGAC |  |  |

**Supplementary Table 2. Annotation of *E. faecium* EF332 genome.**

**Supplementary Table 3. Prediction of genomic islands in *E. faecium* EF332 genome.**

**Supplementary Table 4. Daptomycin susceptibility tests of constructed strains.**

| **Strain** | **MIC (µg/mL)** |
| --- | --- |
| EF332-*cls* | 16 |
| EFDO-*cls* | 8 |
| EF332-*gdpD* | 8 |
| EFDO-*gdpD* | 8 |
| 29212-pDL278 | 8 |
| ATCC 29212 | 1 |

**
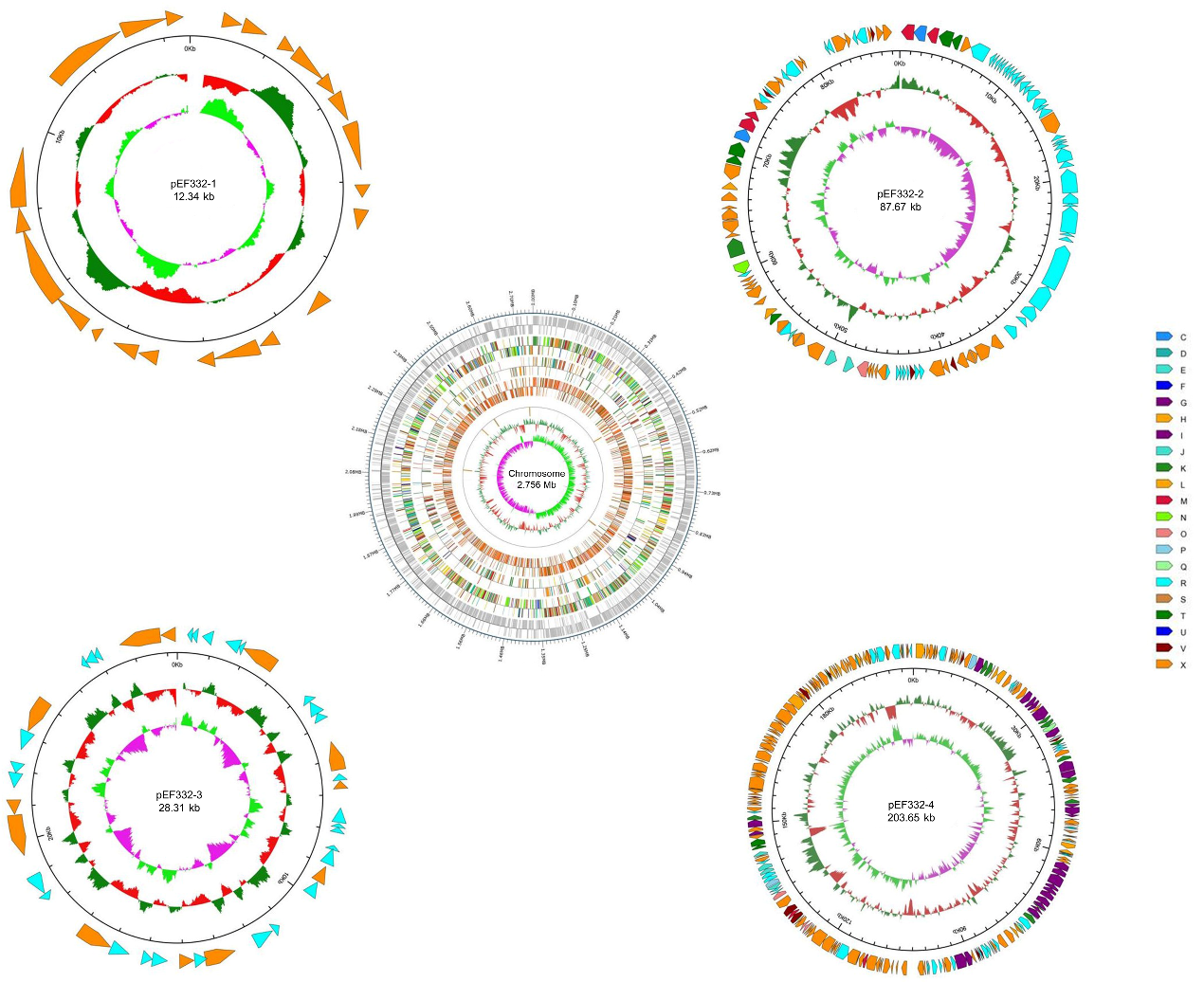
**

**Supplementary Figure 1. Genome maps of *E. faecium* EF332 chromosome and plasmids.** For chromosome, from outside to inside, each circle represents: coordinates of genome sequence position, coding gene, gene functional annotation results (COG, KEGG, GO), ncRNA, GC content, GC skew values. For plasmids, from the outside to the inside, each circle represents: gene functional annotation results by COG (arrow clockwise represents positive encoding chain), coordinates of genome sequence position, GC content, GC skew values. Arrow colors indicate COG categories.

**Reference:**

Kim, J. Y., and Lee, J. L. (2014). Multipurpose assessment for the quantification of *Vibrio spp*. and total bacteria in fish and seawater using multiplex real-time polymerase chain reaction. *J. Sci. Food Agric.* 94, 2807–2817. doi:10.1002/jsfa.6699.
