# Supplementary figures and images for: New Mutations in cls Lead to Daptomycin Resistance in a Clinical Vancomycin- and Daptomycin-Resistant Enterococcus faecium Strain
Source: Front Microbiol. 2022 Jun 21;13:896916. doi: 10.3389/fmicb.2022.896916 (PMC9253605; doi:10.3389/fmicb.2022.896916)

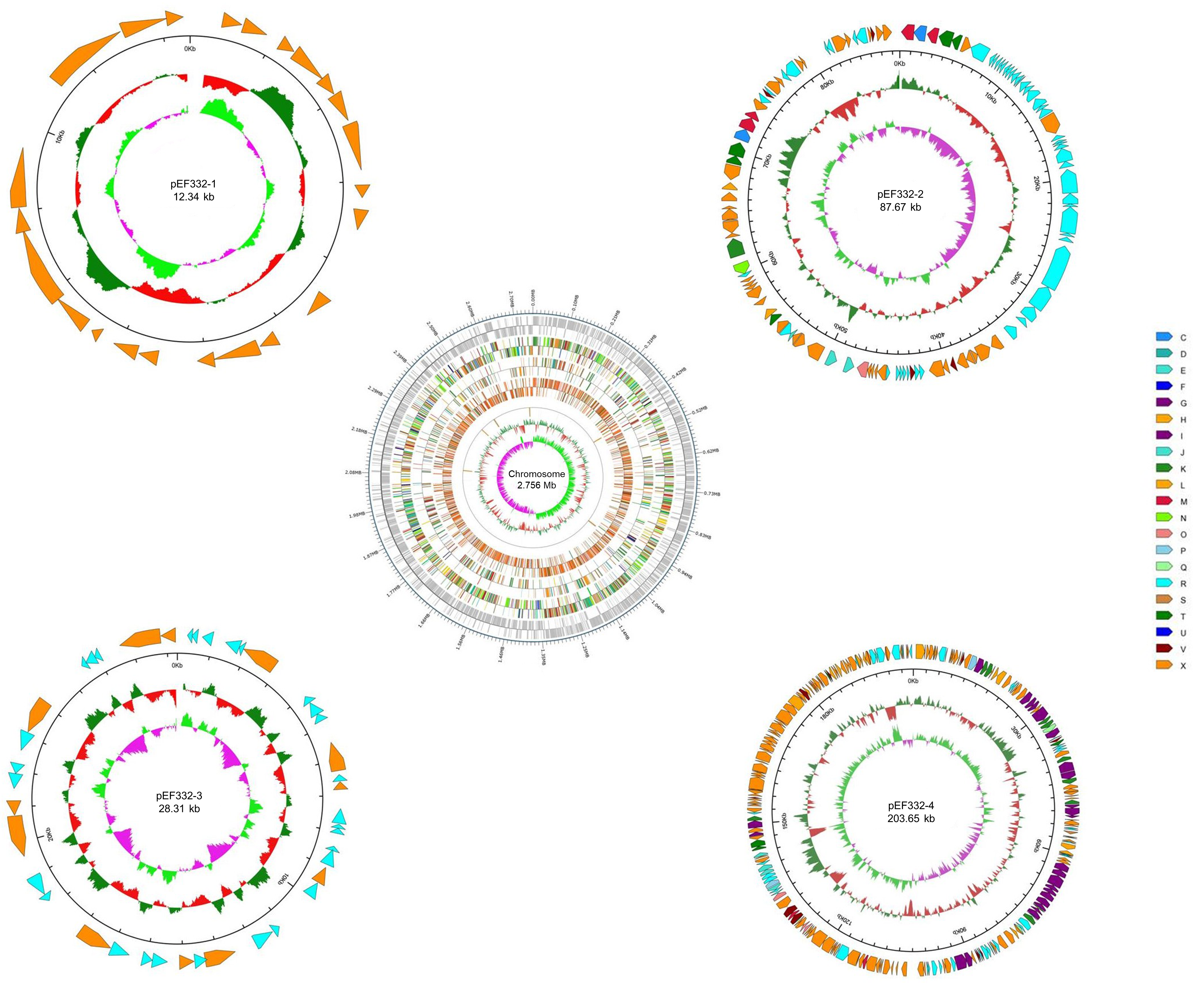

Supplement: Supplementary file 4 [file Image_1.TIF]
